# Metabolomic Profiles Delineate Signature Metabolic Shifts during Estrogen Deficiency-Induced Bone Loss in Rat by GC-TOF/MS

**DOI:** 10.1371/journal.pone.0054965

**Published:** 2013-02-07

**Authors:** Bo Ma, Jiannan Liu, Qi Zhang, Hanjie Ying, Jiye A, Jianguo Sun, Di Wu, Yonglu Wang, Jing Li, Yinhui Liu

**Affiliations:** 1 School of Pharmaceutical Sciences, Nanjing University of Technology, Nanjing, People’s Republic of China; 2 Department of Respiration, Jiangsu Geriatric Hospital, Nanjing, People’s Republic of China; 3 School of Life Science and Pharmaceutical Engineering, Nanjing University of Technology, Nanjing, People’s Republic of China; 4 Key Laboratory of Drug Metabolism and Pharmacokinetics, China Pharmaceutical University, Nanjing, People’s Republic of China; 5 Laboratory for Systems Biology, Division of Clinical Pharmacology and Therapeutics, The Children’s Hospital of Philadelphia, Philadelphia, Pennsylvania, United States of America; 6 Kinetic Modeling and Simulation (KMAS) Core, Institute of Translational Medicine and Therapeutics, University of Pennsylvania, Philadelphia, Pennsylvania, United States of America; Hosptial Infantil Universitario Niño Jesús, CIBEROBN, Spain

## Abstract

Postmenopausal osteoporosis is a complicated and multi-factorial disease. To study the metabolic profiles and pathways activated in osteoporosis, Eight rats were oophorectomized (OVX group) to represent postmenopausal osteoporosis and the other eight rats were sham operated (Sham group) to be the control. The biochemical changes were assessed with metabolomics using a gas chromatography/time-of-flight mass spectrometry. Metabolomic profile using serial blood samples obtained prior to and at different time intervals after OVX were analyzed by principal component analysis (PCA) and Partial least squares-discriminant analysis (PLS-DA). The conventional indicators (bone mineral density, serum Bone alkaline phosphatase (B-ALP) and N-telopeptide of type I collagen (NTx) of osteoporosis in rats were also determined simultaneously. In OVX group, the metabolomics method could describe the endogenous changes of the disease more sensitively and systematically than the conventional criteria during the progression of osteoporosis. Significant metabolomic difference was also observed between the OVX and Sham groups. The metabolomic analyses of rat plasma showed that levels of arachidonic acid, octadecadienoic acid, branched-chain amino acids (valine, leucine and isoleucine), homocysteine, hydroxyproline and ketone bodies (3-Hydroxybutyric Acid) significantly elevated, while levels of docosahexaenoic acid, dodecanoic acid and lysine significantly decreased in OVX group compared with those in the homeochronous Sham group. Considering such metabolites are closely related to the pathology of the postmenopausal osteoporosis, the results suggest that potential biomarkers for the early diagnosis or the pathogenesis of osteoporosis might be identified via metabolomic study.

## Introduction

Postmenopausal osteoporosis is a skeletal condition associated with reduced bone mineral and bone strength, involved in millions of people worldwide, especially those with pathological fracture. Osteoporosis is also called the “silent disease” in clinics because majority of people don’t know they have got osteoporosis until it has progressed and diagnosed at the point of fracture, most frequently occurred in the hip, wrist or spine, and the fracture often causes dangerous conditions and leads to deformity, and even death.

Bone mineral density (BMD) as a “gold standard” has been used in osteoporosis for a long time [1], [2]. BMD test can indicate bone density at the normal, relatively low or osteoporotic levels, and predict the risk of fracture at the certain points. However, alterations in bone mineral density are slow in the dynamic disease progress of osteoporosis. Recently, Gourlay et al [3] attempted to standardize the bone-density testing interval (The BMD testing interval was defined as the estimated time for 10% of women to make the transition to osteoporosis before having a hip or clinical vertebral fracture, with adjustment for estrogen use and clinical risk factors.) and transition process to osteoporosis in elder women. Their data indicated that the bone-density testing interval for women with normal bone density or mild osteopenia as well as advanced osteopenia are 15, 5 and 1 year, respectively. As a clinical biomarker, bone mineral density has the disadvantages of slow change and low sensitivity, even frequent BMD testing is unlikely to improve the prediction of fracture and osteoporosis. For this reason, simple, sensitive and specific biomarkers are needed to be discovered, validated and applied for early diagnose of postmenopausal osteoporosis in clinic.

An association between an imbalance of bone formation and bone resorption was identified in pathological study on bone loss. Specific biochemical indicators for bone turnover, including bone formation markers (B-ALP; Osteocalcin et al), and bone resorption markers (NTx; Tartrate-resistant acid phosphatase-5b, TRCAP-5b; and Carboxy-terminal collagen crosslinks, CTX etc), might be used as index for disease progression of osteoporosis[4]–[6]. These sensitive and validated biochemical markers can offer an alternative to well-accepted BMD test to monitor disease progression of osteoporosis and therapeutic treatment [7], [8]. The disadvantage of the biochemical markers is that they only reflect the alteration of bone formation or bone resorption, while the incidence of osteoporosis is attributed to the dual outcomes of bone formation and resorption.

Metabolomics as an important component of systems biology, including genomics, transcriptomics and proteomics, provide a wide spectrum of information on the biochemical finger print in cell, tissue or organism levels to elucidate novel mechanisms by detecting and comparing small-molecule metabolite profiles under difference conditions [9]. Metabolomics is the endpoints of genotype functions and biochemical phenotype in body. Metabolic profiles detected by metabolomics in different conditions are linked closely to functions alteration in body [10]. Biomarkers obtained by metabolomics are more sensitive to disease etiology and progression compared with those obtained by proteinomics and genomics [11], [12]. Metabolomics has been used in the early detection and diagnosis of disease progression and provided prognostic biomarkers as novel therapeutic targets [13]–[16]. Postmenopausal osteoporosis is known as a complex disease, and many pathophysiologic factors involve in its occurrence and progression, including estrogen receptor [17], OPG/RANK/RANKL system [18], inflammatory factor [19] and oxidative stress [20]. Considering there is no sensitive and specific biomarker indicating the pathogenesis of osteoporosis from a holistic point of view so far, metabolomics study might provide suitable approaches to investigate osteoporosis on disease etiology and progression, and identify a cluster of biomarkers of postmenopausal osteoporosis.

In our laboratory, metabolomic approach by using GC-MS or GC-TOF/MS has been successfully applied to the study of hypertension [21], hyperlipemia [22], chronic myeloid leukemia [23], colon carcinoma, and professional athletes [24] in serum or urine from rat or human. Metabolomic study on estrogen deficiency-induced obesity was performed recently and a series of biomarkers was identified to relate to estrogen deficiency [25]. Based on our previous work, the metabolic alterations occurred in osteoporosis induced by estrogen deficiency are examined by using GC-TOF/MS. The goals of study are listed as following. 1) Using GC-TOF/MS to delineate the metabolic profile and monitor the endogenous metabolomics in rat plasma during the process of estrogen deficiency-induced bone loss. 2) Identifying and characterizing specific metabolic pathways served as targets for treatment intervention or as substrates for molecular imaging in postmenopausal osteoporosis. 3) Exploring mechanisms of bone loss induced by estrogen deficiency from the perspective of metabolomics.

## Materials and Methods

### 1. Chemicals and Reagents

Myristic-1,2-^13^C2 acid, 99 atom %^13^C, the stableisotopelabeled compound used as an internal standard (IS),Alkane series (C8–C40), MSTFA (N-methyl-N-trimethylsilyltrifluoroacetamid), and 1% TMCS (trimethylchlorosilane) were obtained from Sigma-Aldrich(St. Louis, MO, USA). HPLC-grade methanol and n-heptane were obtained from Merck (Damstadt, Germany). The reference substance octadecadienoic acid, arachidonic acid, docosahexaenoic acid, isoleucine, valine, leucine, homocysteine, hydroxyproline, tryptophan, cystine, succinic acid, citric acid, malic acid and lysine were purchased from Sigma-Aldrich (St. Louis, MO, USA). Purified water was produced by a Milli-Q system (Millipore, MA, USA).

### 2. Experimental Design

Sixteen virgin Sprague–Dawley female rats with specific pathogen free (SPF) (6 months old, body weight 260±20.0 g) were obtained from the Animal Center of Nanjing Medical University in this study. Rats were housed under controlled conditions, including room temperature kept at 22±1°C, and standard solid food with water ad libitum provided during the experiment. This study was reviewed and approved by the Animal Ethical Committee of Nanjing University of Technology.

After acclimatization for 7 days, all animals were anesthetized with 30 mg/kg pentobarbital sodium (Fluka, Germany), intraperitoneal injection. Eight rats were oophorectomized (OVX group) and the remaining eight rats were sham operated (Sham group). Prior to the experiment and at 9∶00 am on the 3rd, 6th, 9th, 12th, 24th week after operation, 1 mL blood was collected and split equally into 2 tubes to separate serum and plasma. One aliquot whole blood was collected in the tube containing EDTA and placed for 15 min. Then plasma was immediately separated by centrifugation at 2000×g for 10 min at 4 °C. (no food but water ad libitum for 12 h). The serum was used for determinating bone alkaline phosphatase (B-ALP, an indicator of bone formation) and N-telopeptide of type I collagen (NTx) levels by ELISA method (Quantikine, R&D Systems, Minneapolis, MN, USA) following the manufacturer’s instruction with 6.7% intra- and 8.4% inter-assay variabilities for B-ALP as well as 7.2% intra- and 5.7% inter-assay variabilities for NTx, respectively. The plasma sample was used for metabonomic study. All samples were stored at −80°C until analysis. BMD of the lumbar vertebrae (L1–L5) and left femur from anesthetized rats was measured (isoflurane) by dual-energy X-ray absorptiometry (GE, Lunar ProdigyTM) at the 12^th^ 24^th^ week post-operation. BMD was calculated by dividing the measured area by the bone mineral content (BMC), as previously reported [30]. Baseline of BMD was determined 1 week before surgery. All rats were weighed prior to surgical operation and monitored weekly post surgical operation. Uterine tissues were removed and weighed immediately to calculate uterus indexes by dividing the uterus weight by the body weight after rats were sacrificed. Meanwhile, the serum level of estradiol was analyzed immediately by ELISA method following the ELISA kit manufacturer’s instruction.

### 3. Metabolomic Research

#### 3.1 Sample preparation

A 50 µL aliquot of plasma in an eppendorf tube was spiked with 200 uL methanol containing internal standard myristic-1, 2-^13^C_2_ acid (12.5 µg/ml) to deproteinize. After the mixture was vortexed for 3 min, the mixture was centrifuged for 10 min at 12000×g at 4°C. One hundred microliter of the supernatant was transferred into a GC vial, followed by being evaporated to dryness under vacuum in speedvac concentrator (Thermo Fisher Scientific, Asheville, USA). Subsequently, The residue was reconstituted in 30 µl methoxyamine in pyridine (15 mg/ml) and vortexed for 2 min. Methoximation reaction was performed at room temperature for 16 h, before trimethylsilylation for 1 h by adding 30 µl MSTFA with 1% TMCS as catalyst. Finally, the solution was vortexed for 30 s after methyl myristate in heptane (30 µg/ml), the external standard, was added to the GC vial. One microliter of solution was used for GC-TOF/MS analysis.

#### 3.2 GC-TOF/MS analysis

One microliter of the derivatized sample was injected into an Agilent 6980 GC equipped with an Agilent 7683 Series autosampler (Agilent, Atlanta, GA, USA).Chromatographic separation was conducted on a fused-silica capillary column (10 m×0.18 mm ID) chemically bonded with a 0.18 µm DB5-MS stationary phase (J&W Scientific, Folsom, CA, USA). Helium was served as carrier gas through the column. Mass spectra were detected and obtained by using Pegasus III TOF/MS (Leco).The gas flow rate through the column was 1 mL/min. The column initial temperature was kept at 70°C for 2 min. Temperature was increased from 70°C to 310°C at a rate of 20°C/min and held for 2 min. The transfer line temperature and ion source temperature were controlled at 250°C and 200°C, respectively. Ion source voltage and current: 70 eV at a current of 3.0 mA. The MS data were acquired in scan mode over the range between m/z 50 and 800 at a rate of 20 spectra/s, the acceleration voltage was turned on after a solvent delay of 170 s and the acceleration voltage was turned on at1,650 v [26], [27].

#### 3.3 Data collection and processing

All GC/MS data was processed using ChromaTOF (version 3.25) software (Leco). Peak width in automatic peak detection and mass spectrum deconvolution were set to 2 s. Peaks with signal-to-noise (S/N) ratios lower than 30 were rejected. To obtain accurate peak areas for IS and specific peaks/compounds, one quant mass were specified for each peak and the data were reprocessed according to previous report, and each peak area was normalized using IS before multivariate data analysis. All compounds were identified and assigned by comparison of mass spectra and retention index of all detected compounds with the authentic reference standards and those in the NIST library 2.0 (2005), Wiley library, and in-house mass spectra library database established by the Key Laboratory of Drug Metabolism and Pharmacokinetics in China Pharmaceutical University [24], [28].

Before multivariate data analysis, the peak area ratio of each compound to a corresponding internal standard was calculated as the response. Then, the data were imported to SIMCA-P v11.5 software (Umetrics AB, Sweden) for multivariate data analysis. Partial least squares-discriminant analysis (PLS-DA) and Principal component analysis (PCA) were used for modeling metabonomic data from Sham group and OVX group.

Data obtained from BMD, body weight, commercial assay kits were presented as the mean ± SD. The data were analyzed using one-way analysis of variance followed by a least significant difference (LSD) test at P<0.01 and P<0.05.

## Results

### 1. Body Weight, Uterine Index and E2

The representative change tendency of body weight during the whole experiment period is listed in Table 1. The body weights of rats in OVX group were significantly higher than those in Sham group starting at 4^th^ week post operation. Estrogen deficiency caused atrophy of uterine tissue, and serum E2 levels of rats in OVX group significantly deceased at the end of experiment compared with those in Sham group (shown in Table 1). Both data indicated the successful surgical procedure.

**Table 1:** Body weight, BMD, Uterine index, and Serum estradiol from the OVX and the Sham rats.

Parameters		The Sham group	The OVX group
Initial body weight (g)		252.62±11.47	250.72±16.35
Final body weight (g)		326.43±27.66	403.51±32.76**
Initial BMD(g/cm2)	*Femur*	0.210±0.012	0.211±0.010
The 12^th^ week BMD(g/cm2)		0.224±0.019	0.201±0.010*
The 24^th^ week BMD(g/cm2)		0.236±0.017	0.207±0.015*
Initial BMD(g/cm2)	*lumbar vertebrae*	0.202±0.011	0.201±0.015
The 12^th^ week BMD(g/cm2)		0.213±0.016	0.188±0.017*
The 24^th^ week BMD(g/cm2)		0.221±0.022	0.185±0.012**
Uterine index (mg/g)		2.312±0.252	0.532±0.124**
Serum estradiol (pk/mL)		35.53±6.45	13.53±5.47**

Values with a superscript are significantly different from the Sham group (*P<0.05, **P<0.01).

### 2. Bone Mineral Density Measurement

As shown in Table 1, at the12^th^ week and 24^th^ week after OVX, using DEXA analysis, significant decrease in BMD values for both the femur (P<0.05 for 12 weeks and P<0.05 for 24 weeks) and the lumbar vertebrae (P<0.05 for 12 weeks and P<0.01 for 24 weeks) were observed in rats in OVX group compared with those in Sham group.

### 3. Biochemical Studies

**Figure 1:**
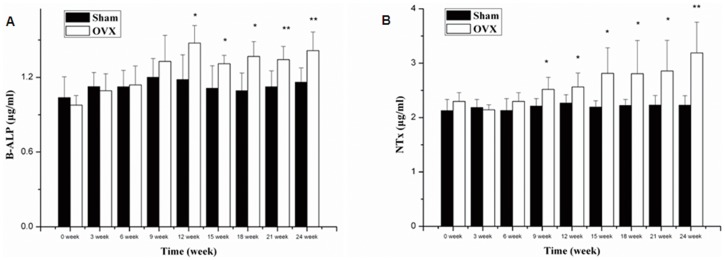
Biochemical parameters in serum. Biochemical parameters in serum from the Sham group and the OVX group over the whole experiment period. The values represent as the Means±SD. N = 8. (*P<0.05, **P<0.01 vs Sham).

Estrogen deficiency resulted in gradual increase in serum B-ALP and NTx in OVX rats after surgery. B-ALP and NTx of rats in OVX group significantly increased since the 12^th^ week (P<0.05) and 9^th^ week (P<0.05) post operation, respectively, compared with those in Sham group(shown in Figure 1A and 1B). The results indicated that OVX rat had the characteristic of bone high turnover rate according to the previous reports [29], [30].

### 4. Metabolic Changes in Plasma Samples by GC-TOF/MS

**Figure 2:**
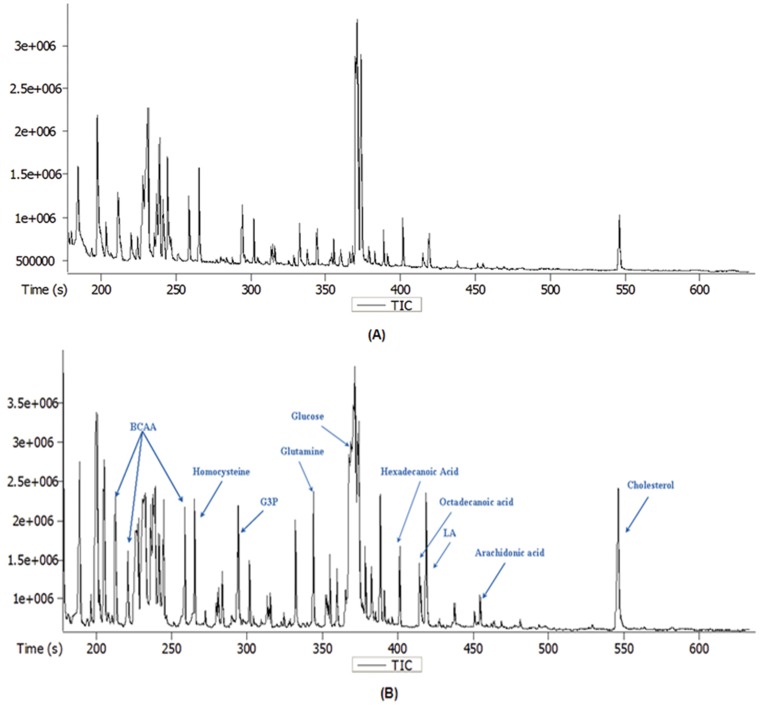
Typical GC-TOF/MS total ion chromatograms of rat plasma. Typical GC-TOF/MS total ion chromatograms of rat plasma obtained from the Sham group (A) and the OVX group (B).

**Figure 3:**
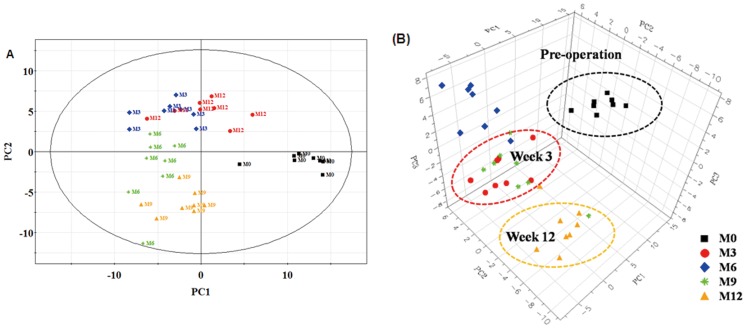
PLS-DA score plots and 3D plots. Partial least squares projection to latent structures and discriminant analysis (PLS-DA) score plots (A: PC1/PC2) and 3D plots (B: PC1/PC2/PC3) of the OVX group over the process of bone loss. X-axis, Y-axis and Z- axis were labeled with PC1 (the first principal component), PC2 (the second principal component), PC3 (the third principal component) respectively. Letters M denotes the OVX group. Numbers 0–12 represents different time points during the experiment: 0, before experiment; 3, week 3; 6, week 6 et al. M3 means the point from the sample in the OVX group at the 3^rd^ week after the experiment. One data point stands for one subject: for instance, M3 and M6 mean the point from the sample in the OVX control rats at the 3rd and 6th week after surgery.

Distinct differences of the major metabolites levels between the OVX and Sham groups were observed in GC-TOF/MS chromatogram, shown in Figure 2A and Figure 2B. PCA (data not shown) and PLS-DA were performed to further discern the presence of inherent similarities in chromatographic profiles. The PLS-DA score plots (Figure 3A) and 3D plots (Figure 3B) of the metabolites showed the metabolomic movement during the periods of osteoporotic progression including pre-operation, the 3^rd^, 6^th^, 9^th^ and 12^th^ week post operation (these five time points were marked as M0, M3, M6, M9, and M12 in the figures respectively). The distinguished separations among M0, M3, M6, M9, and M12 were observed clearly. Postoperative plasma metabolomic profiles were observed to be distinguished from preoperative samples starting at the 3^rd^ week. However, classical pathological indexes including BMD, plasma B-ALP and NTx did not appear significant changes still the 9^th^ weeks.

In PLS-DA, the R2X, R2Y and Q2(cum) parameters were used for the model evaluation, representing the explanation, fitness and prediction power respectively. (R2X is the percentage of all GC/TOF-MS response variables explained by the model. R2Y is the percentage of all observation or sample variables explained by the model. Q2 is the percentage of all observation or sample variables predicted by the model). In the PLS-DA score plots (Figure 3A), two principal components (PC1 and PC2) were calculated with the R2X, R2Y and Q2 parameters of 0.449, 0.848 and 0.56, respectively.

**Figure 4:**
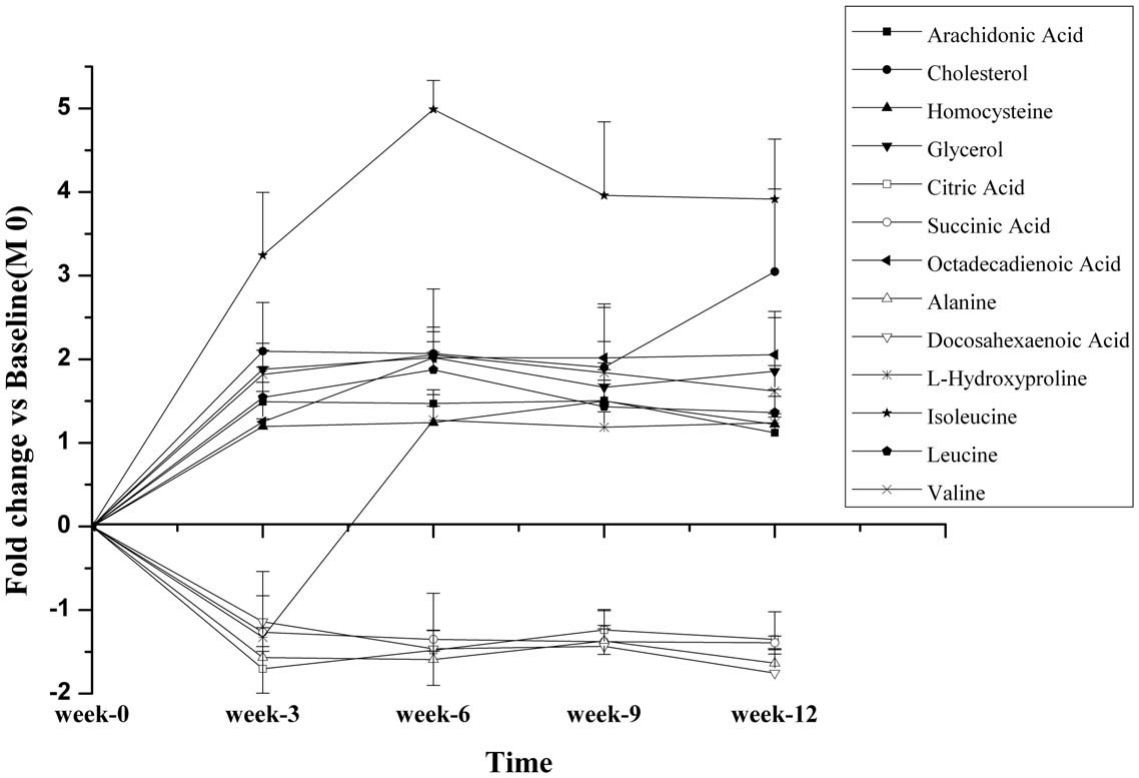
Kinetics of metabolic changes in the OVX plasma. Kinetics of metabolic changes in the OVX plasma over the 12 week post operation.

**Figure 5:**
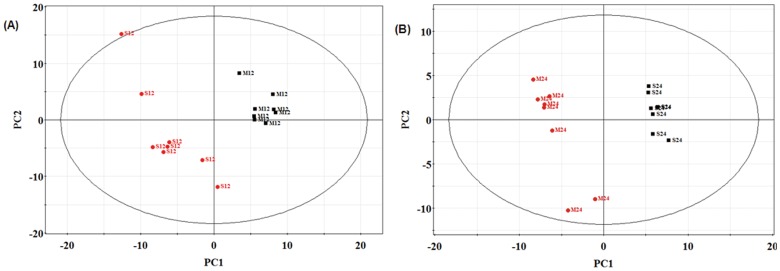
PLS-DA score plots of plasma samples. (A) PLS-DA score plots of OVX rats and sham rats at the 12^th^ weeks post-surgery; (B) PLS-DA score plots of OVX rats and sham rats at the 24^th^ weeks post-surgery; (C) PLS-DA score plots of OVX rats between the 12^th^ weeks and 24^th^ week post surgery. Letters M and S denotes the OVX group and the Sham group, respectively. Numbers 12 and 24 represents different time points during the experiment: 12, week 12; 24, week 24.

Kinetics of metabolic changes in rat plasma before and after bilateral oophorectomy was shown in Figure 4. Plasma levels of alanine, citric acid, succinic acid, and docosahexaenoic acid decreased significantly while homocysteine, branched-chain amino acids (leucine, isoleucine and valine), fatty acids (arachidonic acid and octadecadienoic acid) and cholesterol increased significantly after OVX operation compared with those before operation. It’s noted that the level of hydroxyproline was low at the 3^rd^ week post operation, but unexpectedly and significantly increased at the 6^th^ week. Figure 5C described the progression of the osteoporosis analyzed by PLS-DA, that plasma samples at the 12^th^ week were well discriminated from those at the 24^th^ week in OVX group.

**Figure 6:**
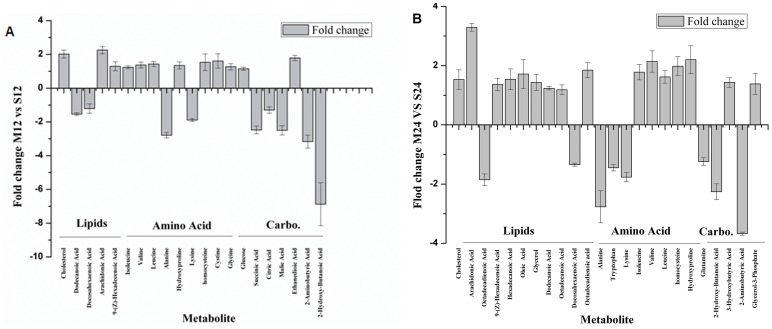
Relative changes of plasma metabolite levels. Relative changes of plasma metabolite levels in the sham group versus the OVX group at the 12^th^ weeks and 24^th^ weeks post surgery.

The metabolic profiles of OVX and Sham groups at the same period were also analyzed and compared by PLS-DA. It’s shown in Figure 5A and Figure 5B that metabolic profiles of OVX and Sham groups separated distinctly at the 12^th^ week and 24^th^ week post operation. Two principal components were calculated with the set of R2X, R2Y and Q2 parameters as 0.335, 0.946 and 0.714, and 0.325, 0.996 and 0.582 respectively. Metabolic changes were listed in Figure 6 and Table 2. In detail, the plasma level of arachidonic acid in OVX group elevated at 12^th^ week (2.5-fold, *P* = 0.0050) and 24^th^ week (3.29-fold, *P* = 0.0023), compared with that in the homeochronous Sham group. Omega-6 fatty acids- octadecadienoic acid also elevated at 12^th^ week (1.19-fold, *P* = 0.5302) and 24^th^ week (1.85-fold, *P* = 0.0046). Homocysteine (1.53-fold at the 12^th^ week, *P* = 0.042; 1.98-fold at the 24^th^ week *P* = 0.0442) and Hydroxyproline (2.2-folds the 12^th^ week, *P* = 0.0060; 2.43-folds at the 24^th^ week *P* = 0.0053) concomitantly enhanced in plasma. However, the levels of omega-3 fatty acids (docosahexaenoic acid) in OVX group decreased (−1.22-fold P = 0.3508, −1.54-fold P = 0.0012) compared with those in the homeochronous Sham group. Elevated levels of branched-chain amino acids (leucine, isoleucine and valine), cholesterol, tryptophan, cystine and glycerol-3-phosphate were also found at the 12^th^ week and 24^th^week. Interestingly, a portion of metabolite changes were different in the12^th^ week and 24^th^ week. The levels of intermediate metabolites of TCA (succinic acid, citric acid and malic acid) significantly decreased at the 12^th^ week, then gradually returned to the normal level at the 24^th^ week.

**Table 2:** Fold changes and P value of endogenous metabolites detected by GC/TOF-MS.

Metabolites	Retentionindex	Biologicalrole	12 weeksaftersurgery		24 weeks after surgery	
			Foldchange	*P*	Fold change	*P*
Cholesterol	3178.2	a sterol and a lipid found in the cell membranes	2.02	0.047	1.53	0.006
Octadecadienoic acid	2216.4	an unsaturated n-6 fatty acid and essential fatty acid	1.19	0.533	1.85	0.005
Arachidonic acid	2375.2	a polyunsaturated, essential fatty acid and precursor ofprostaglandins, thromboxanes, and leukotrienes	2.25	0.005	3.29	0.002
Oleic acid	2215.3	An unsaturated fatty acid	1.09	0.793	1.72	0.013
9-(Z)-Hexadecenoic Acid	2029.7	an unsaturated fatty acid, a common constituentof the glycerides of adipose tissue	1.29	0.031	1.36	0.009
Hexadecanoic Acid	2049.2	a saturated fatty acid	−1.16	0.401	1.54	0.010
Octadecanoic Acid	2245.6	a saturated fatty acid	−1.10	0.340	1.18	0.047
Docosahexaenoic Acid	2566.9	an omega-3 fatty acid and associated with the heart disease	−1.22	0.350	−1.54	0.001
Glycerol	1282.4	an important component of triglycerides and of phospholipids, energy metabolism	1.18	0.362	1.43	0.013
Isoleucine	1305.8	Essential branched-chain aliphatic amino acid associated with energy metabolismand blood sugar regulation	1.24	0.012	1.78	0.018
Valine	1230.8		1.37	0.042	2.14	0.010
Leucine	1285.2		1.43	0.035	1.62	0.004
homocysteine	1437.9	sulfur-containing amino acid, associated with cardiovasculardisease, Alzheimer’s disease and fracture	1.53	0.042	1.98	0.044
L-Hydroxyproline	1522.4	a common non-proteinogenic amino acid and Paget’s disease	2.20	0.006	2.43	0.005
Tryptophan	2244.0	an essential amino acid which is the precursor of serotonin	1.03	0.780	−1.44	0.001
Glutamine	1608.8	energy metabolism, nitrogen balances	1.01	0.969	−1.24	0.022
Cystine	1433.9	immune function and the synthesis of glutathione	1.61	0.030	1.44	0.226
Glycine	1438.4	a simple, nonessential amino acid	1.27	0.039	1.15	0.837
Alanine	1107.5	An important participant and regulator in glucose metabolism	−2.79	0.005	−2.77	0.017
Succinic Acid	1323.2	citric acid cycle, Glucose metabolism	−2.47	0.022	−1.14	0.814
Citric Acid	1844.1		−1.30	0.043	−1.21	0.071
Malic Acid	1502.8		−2.50	0.042	−1.26	0.797
Glucose	1935.6	a monosaccharide containing six carbon atoms	1.15	0.010	1.06	0.318
L-Lysine	1720.8	an essential amino acid	−1.89	0.011	−1.76	0.002
Ethanedioic Acid	1144.9	glyoxylic acid or ascorbic acid metabolism	1.79	0.004	1.25	0.302
2-Hydroxy-Butanoic Acid	1137.3	an organic acid that is involved in propanoate metabolism	−6.88	0.012	−2.26	0.007
Dodecanoic Acid	1656.1	a component of triglycerides	−1.54	0.023	1.24	0.026
2-Aminobutyric Acid	1185.5	a key intermediate in the biosynthesis of ophthalmic acid or ophthalmate.	−3.16	0.012	−3.67	0.003
3-Hydroxybutyric- Acid	1172.8	ketone body	−1.24	0.425	1.43	0.010
Glycerol-3-Phosphate	1787.1	a chemical intermediate in the glycolysis metabolic pathway	1.10	0.320	1.38	0.044

## Discussion

A rat model of estrogen deficiency-induced bone loss was chosen to simulate postmenopausal osteoporosis. Comprehensive analysis of changes in the plasma metabolic profile was performed to determine step-wise disease progression from normal conditions to estrogen deficiency and eventually to bone loss.

As shown in Fig. 3A and Fig. 3B, distinct changes were observed from GC-TOF/MS chromatograms in OVX and Sham groups. Further investigations in biomarkers identification were performed and significant differences were shown in Table 2. The alteration of these molecules including amino acids, carbohydrates, fatty acids and cholesterol suggested that they might play certain roles in the etiology of postmenopausal osteoporosis. The early detection of metabolic changes linked to the onset of disease suggested that metabolic alterations might precede BMD and other related diagnostic indexes, and metabolomics might be a sensitive technology to diagnose osteoporosis superior to the classical pathology indexes.

Metabolite fingerprinting obtained from metabolomics could be applied not only to early diagnosis of diseases, but also to identify the causes of disease via biomarker index. Metabolic biomarkers identified by GC-TOF/MS would be beneficial to systemically understand the mechanisms of postmenopausal osteoporosis and to design the target points of anti-osteoporosis drugs. A great many endogenous molecules including fatty acids, cholesterol, amino acids, and carbohydrates exhibited significant difference in OVX group compared with those in Sham group. In the process of bone loss, the metabolism of fatty acids (octadecadienoic acid, arachidonic acid, oleic acid, docosahexaenoic acid, dodecanoic acid, aminomalonic acid, hexadecanoic acid), homocysteine, branched-chain amino acids (BCAA) and cholesterol were also significantly changed in our research.

Significant increase of the fatty acid levels in the process of bone loss are particularly noted in our metabolomic study. Arachidonic acid (AA, 20∶4n-6), the n-6 long chain polyunsaturated fatty acid (LCPUFA), is a precursor of prostaglandins, thromboxanes, and leukotrienes. AA play an important role in the regulation of the metabolism on lipid protein, the body’s inflammatory response, muscle growth, blood rheology, elasticity of blood vessels, white blood cell function and platelet activation[31]–[33]. Meanwhile, AA also affects the bone remodeling process. AA was found to promote osteoclastogenesis by stimulating RANK-L expression and inhibiting OPG secretion by osteoblasts. Its metabolites (prostaglandin and leukotrienes) also affect the bone formation and bone reabsorption. Coetzee et al, indicated that AA inhibited the secretion of OPG by osteoblasts, reduce the OPG/RANKL ratio and may increase osteoclastogenesis and prostaglandin E2 (PGE2), a pro-inflammatory lipid mediator derived from arachidonic acid by the activity of cyclooxygenase (COX), has similar effects on MG-63 and MC3T3-E1 osteoblast-like cells [34]. Moreover, leukotrienes, the fatty signaling molecules derived from arachidonic acid, and particularly IL-1 and IL-6 (potent bone-resorbing cytokines), have been implicated in bone remodeling and disease–specifically in osteoporosis and rheumatoid arthritis[35]–[37]. Abnormal metabolism pathway of AA due to estrogen deficiency might act as an important factor in the pathogenesis of osteoporosis based on our study data.

In contrast to AA, the levels of oleic acid and docosahexaenoic acid (DHA, 22∶6n-3) decreased in plasma during bone loss induced by estrogen deficiency. An augmentation of the n-6/n-3 polyunsaturated fatty acid ratio was shown to lead to increased bone loss in both animal [38] and human [39]. The elevated levels of the n-6 polyunsaturated fatty acids (AA, Octadecadienoic acid) and decreased levels of n-3 polyunsaturated fatty acids (Docosahexaenoic acid) also supported these results.

In our previous study, significant elevated levels of plasma cholesterol were found in estrogen deficiency-induced obesity in ovariectomized rats [25]. Enhanced levels of cholesterol and increased body weight were also observed in bone loss rats induced by estrogen deficiency in the current study. Enhanced cholesterol uptake and biosynthesis or inhibition of cholesterol oxidation might explain the reason why estrogen deficiency increased total cholesterol accumulation in plasma. And, the negative relationship between estrogen and cholesterol might be an important index in the process of the menopause for the high incidence of cardiovascular disease, dementia, Alzheimer’s disease, type 2 diabetes and osteoporosis in menopausal women [40]. Cholesterol and its metabolites would suppress the functional activity of osteoblasts and thereby induce reduced bone mineralization to decrease bone formation [41]. By the same token, high cholesterol diet increases the risk of osteoporosis, possible via inhibiting the differentiation and proliferation of osteoblasts in rats, as You et al observed [42]. At present, the drug treatment against osteoporosis can prevent bone loss, as well as lowering cholesterol levels. Arzoxifene, a new selective estrogen receptor modulator (SERM), maintained bone formation, prevented bone loss by decreasing osteoclast number following by reducing serum cholesterol in OVX rats in Ma and co-workers’ study [43]. Statins, the enzyme HMG-CoA reductase inhibitors, initially used for treating hyperlipidemia, were recently observed not only to prevent fractures, but also trigger significant bone re-growth. From the mechanistic point of views, statins were found to inhibit mevalonate synthesis, prevent the synthesis of cholesterol and indirectly further alter osteoblasts and osteoclast activity, its direct biologic action also affect on osteoblast activity by stimulating gene expression of bone morphogenetic protein-2 [44], [45].

For the amino acids metabolism, above all, homocysteine levels significantly increased in OVX group compared to the Sham group. Homocysteine is a non-protein amino acid biosynthesized from methionine by the removal of its terminal Cε methyl group and recycled into methionine or converted into cysteine with the aid of B-vitamins. Homocysteine is still considered as part of a screen for people at the high risk of heart attack or stroke in clinics. A negative relationship between exogenous estrogen change and homocysteine levels was observed in post-menopause women, and estrogen blocking homocysteine-induced endothelial dysfunction in porcine coronary arteries was reported by Todd [46]. In our study, a gradually increasing homocysteine level followed by decreasing estrogen concentrations was found in plasma after ovaries removed. A positive correlation of homocysteine and osteoporosis was also observed in metabolomic profiles. The research led by McLean and Jacques indicated homocysteine could be a predictive factor for osteoporotic fracture in older persons [47]. For the correlation of homocysteine levels and osteoporosis, recent *in vitro* studies performed in mouse bone marrow cells have suggested that homocysteine directly stimulates osteoclast formation and activity (upregulated TRACP+ multinucleated cells and TRACP activity) by inducing p38 MAPK activity and intracellular reactive oxygen species (ROS) generation, which is a risk factor for osteoporosis and fracture [48]. An increased homocysteine level in plasma was mainly attributed to the lack of estrogen caused in postmenopausal osteoporosis. In terms of specific biochemistry pathways, estrogen influenced metabolism transformation between methionine and homocysteine, especially interfering with the transsulfuration pathway. Estrogen could enhance cystathionine-β-synthase activity and directed homocysteine metabolism to form cysteine and glutathione to eliminate homocysteine and prevent its accumulation, that may act as a key aspect in elucidating this phenomenon [49].

It suggested that homocysteine could be a potential biomarker in diagnosis, and treatment against postmenopausal osteoporosis. Some anti-osteoporosis drugs such as strontium ranelate [50], raloxifene [51] etc, could inhibit increased homocysteine levels in plasma induced by estrogen deficiency and reduce the risk of osteoporosis. Therefore, homocysteine enhancing excretion of amino acids might be a strong indicator in both post-menopause and osteoporosis, and could be a potential target point or a key factor of upper stream controller for anti-osteoporosis treatment.

Hydroxyproline is a major component of the protein collagen and plays key role for collagen stability. Some research suggested that increased serum and urine levels of hydroxyproline had been demonstrated in Paget’s disease bone [52], [53]. Meanwhile, mean plasma levels of branched-chain amino acids (BCAA, leucine, isoleucine, and valine) raised rapidly in the first three week after OVX and remained significantly elevated (p<0.05 versus sham-operated controls) for the entire 24 week. Overall, these observations indicated that increased homocysteine and hydroxyproline levels in OVX rats, besides increased accumulation of BCAA, may be potential biomarkers of postmenopausal osteoporosis.

Estrogen deficiency is the main reason of post-menopause osteoporosis. So, we used OVX rats, which are golden standard models of the post-menopause osteoporosis, to study the estrogen deficiency-induced bone loss and got the metabolomic profiles to delineate signature metabolic shifts during the progress of the estrogen deficiency. On the other hand, estrogen is involved in the regulation of many physiological functions of the body. Estrogen deficiency also induces other physical changes such as obesity. Therefore, on the basis of the current results, the further studies, that could link the metabolites found in this study to the osteoblasts or osteoclasts which are essential to the bone loss, should be carried out to confirm the specific biomarkers on estrogen deficiency-induced bone loss.

### Conclusion

The global metabolomics approach applied in our research demonstrated that estrogen deficiency-induced bone loss resulted in a marked change in the plasma metabolic profile that appeared earlier than the known biomarkers of postmenopausal osteoporosis, such as BMD and serum B-ALP and NTx. In the preclinical osteoporosis model, significant changes of endogenous metabolites and specific metabolites were identified which not only regulated bone remodel effects, but also pointed to the mechanisms of disease etiology and progression. Further efforts should focuse on the validation of our findings and identification of genuine biomarkers from spurious biomarkers through preclinical and clinic study.
